# Role of Matrix Metalloproteinase-2 in the Development of Atherosclerosis among Patients with Coronary Artery Disease

**DOI:** 10.1155/2023/9715114

**Published:** 2023-07-07

**Authors:** Nazirah Samah, Azizah Ugusman, Adila A. Hamid, Nadiah Sulaiman, Amilia Aminuddin

**Affiliations:** ^1^Department of Physiology, Faculty of Medicine, Universiti Kebangsaan Malaysia, 56000, Cheras, Kuala Lumpur, Malaysia; ^2^Centre for Tissue Engineering & Regenerative Medicine, Faculty of Medicine, Universiti Kebangsaan Malaysia, 56000, Cheras, Kuala Lumpur, Malaysia

## Abstract

Coronary artery disease (CAD) is a caused by atherosclerotic plaque buildup in the coronary arteries that supply blood and oxygen to the heart. Matrix metalloproteinase (MMP) is a family of zinc-dependent endopeptidase that is involved in various stages of atherosclerosis as demonstrated in *in vitro* and *in vivo* studies. MMP-2 is associated with both stable and unstable atherosclerotic plaque formation. The current review aimed to identify the role of MMP-2 in atherosclerosis development among CAD patients. Literature search was conducted through four online databases and only studies that were published from 2018 until February 2023 were included. The risk of bias was assessed by using the Newcastle–Ottawa Scale. A total of 10,622 articles were initially identified, and only eight studies that fulfilled the selection criteria were included in this review. The results showed that MMP-2 levels and activity were higher in patients with unstable CAD than those with stable CAD and healthy subjects. There was a significant association between MMP-2 levels and cardiovascular disease with MMP-14 levels, which is a pro-MMP-2 activator. In addition, two single nucleotide polymorphisms of the MMP-2 gene (rs243865 and rs243866) were significantly associated with the development of atherosclerosis. In conclusion, MMP-2 plays a crucial role in the development of atherosclerosis among patients with CAD and could be a potential target for CAD therapy.

## 1. Introduction

Coronary artery disease (CAD) is the commonest type of heart disease involving the formation of plaque in the lumen of coronary arteries. A plaque is a buildup of fatty material that causes a heart attack by constricting the vessel lumen and obstructing blood flow to the heart [1, 2]. CAD is one of the cardiovascular diseases (CVDs) with the highest estimated prevalence (53%) in 2019 compared with other CVDs [3]. Globally, it was estimated that 244.1 million people were living with CAD in 2020 [4]. Countries in Central and South Asia have the highest prevalence and mortality rate of CAD [4].

The biological root of CAD is atherosclerosis. Atherosclerosis begins with infiltration and deposition of cholesterol in the arterial wall, migration of vascular smooth muscle cells (VSMCs) to the intima, and development of fibrous matrix and plaque [5]. Over time, this plaque grows and impairs the blood flow to the heart muscle, subsequently causing angina [6]. There are two types of plaque, namely stable and unstable plaques. Stable plaque is associated with a stable CAD or chronic coronary syndrome (CCS) which usually presents with angina that is relieved by rest. CCS is further divided into two types; obstructive coronary and ischemia with nonobstructive coronary arteries (INOCA). An obstructive coronary present with blockage that is greater than or equal to 50% of the vessel diameter, while INOCA indicates ischemia with less than 50% blockage.

Meanwhile, an unstable plaque is prone to rupture, causing thrombosis to occur. Plaque thrombosis results in a near-complete or complete occlusion of the arterial lumen. This condition is known as unstable CAD or acute coronary syndrome (ACS) [7]. Myocardial infarction with ST-elevation (STEMI), non-ST-elevation (NSTEMI), unstable angina (UA), and myocardial infarction with the nonobstructive coronary arteries (MINOCA) are all examples of ACS [8]. Most ACS patients may experience symptoms such as substernal chest pain, shortness of breath, sweating, nausea, and lightheadedness [9, 10]. About 60%–65% of myocardial infarction cases in young people are caused by plaque rupture [11].

A group of proteolytic enzymes known as matrix metalloproteinases (MMPs) is involved in various stages of atherosclerosis development as demonstrated in *in vitro* and *in vivo* studies [12–18]. MMPs are a family of zinc-dependent endoproteases which are secreted by endothelial cells, VSMCs, fibroblasts, osteoblasts, macrophages, neutrophils, and lymphocytes [19]. MMPs are made up of 28 members which are classified based on their structures and organization of their structural domains [20]. Each MMP contains at least three homologous protein domains; signal peptide, propeptide, and catalytic domains. The signal peptide domain directs MMPs to the secretory pathway, whereas the propeptide domain is removed when MMPs are activated. The zinc-binding region is located in the catalytic domain, which is crucial for MMPs to function as proteases. Additionally, the hemopexin domain interacts with tissue inhibitors of MMPs (TIMP) and is involved in substrate binding [21]. MMP-2 activity is inhibited by TIMP-2, whereas MMP-9 activity is inhibited by TIMP-1 [22].

**Figure 1:**
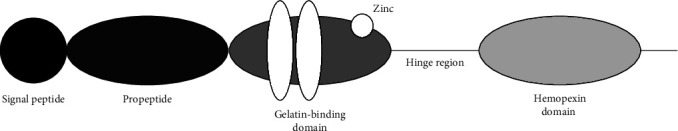
Structure of matrix metalloproteinase-2 (MMP-2).

One of the 28 members of the MMP family is MMP-2. MMP-2 belong to the gelatinase group, which has a gelatin-binding site in their catalytic domain (Figure 1). MMP-2 is associated with both stable and unstable atherosclerotic plaques. During stable atherosclerotic plaque development, MMP-2 is involved in VSMC accumulation in the fibrous cap that shields the plaque. MMP-2 is the first proteinase shown to be responsible for the migration and proliferation of VSMCs [23]. According to a study by Sluijter et al. [24], MMP-2 activity increased in the VSMCs of fibrous-type atherosclerotic plaque, indicating that MMP-2 is strongly linked to the development of stable plaque. Consistent with this, MMP-2 was found to enhance plaque stability by accumulating VSMCs into the fibrous cap of atherosclerotic apolipoprotein-E knockout (ApoE-/-) mice [25].

However, the gelatin-binding site in the MMP-2 catalytic domain made MMP-2 capable of degrading gelatins in the extracellular matrix (ECM) of the fibrous cap enclosing the atherosclerotic plaque, hence promoting plaque instability [21, 26]. Gelatinase is an important enzyme in acute myocardial infarction (AMI) since it can degrade the fibrillar collagen of ECM [27]. According to a study by Zeng et al. [28], atherosclerotic plaque instability in ACS patients may be predicted by high circulating levels of MMP-2. Recently, the activation of the transforming growth factor-beta/suppressor of mothers against decapentaplegic (TGF-/Smad) pathway revealed an increase in MMP-2 levels in rats with myocardial infarction [29]. Meanwhile, the histopathological study showed that MMP-2 was mostly found in fatty streaks and fibroatheromas with hemorrhage and calcification [30]. Since MMP-2 plays a vital part in the progression of atherosclerotic plaque, this review sought to systematically investigate the role of MMP-2 in atherosclerosis development in CAD patients.

## 2. Methodology

This review was conducted in accordance with the Preferred Reporting Items for Systematic Reviews and Meta-Analyses (PRISMA) guideline [31]. PRISMA checklist was reported in Tables 1 and 2.

### 2.1. Search Strategy

The literature search was conducted through four online databases (Ovid, Scopus, Pubmed, and Google Scholar) and only studies that were published from 2018 until February 2023 were included. The keywords used for the search were: (CD) OR (CAD) OR (ischemic heart disease) OR (ACS) OR (coronary atherosclerosis) OR (myocardial infarction) AND (MMP).

### 2.2. Study Criteria

The articles were studied individually by two researchers (NS and AA) centered on the inclusion and exclusion criteria. The inclusion criteria include (1) full-text original articles in English language, (2) studies that reported MMP-2 in atherosclerotic CAD, and (3) clinical studies involving adult human patients with CAD, both male and female, of any ethnicity. While the exclusion criteria were: (1) not original articles, (2) published in languages other than English, (3) studies that not reported MMP-2 in atherosclerotic CAD, (4) articles including reviews, conference abstracts, editorials, newsletters, book, and book chapters, and (5) *in vitro* and *in vivo* studies.

### 2.3. Article Selection and Data Extraction

Three phases of article selection were carried out. Initially, articles were excluded primarily on the title. Then, by analyzing the abstracts, articles that were not relevant to MMPs and atherosclerosis were excluded. In Last, after reading the complete articles, articles that did not meet the inclusion requirements were excluded. For data extraction, two researchers (NS and AA) independently recorded the name of the studies' first author, the study's design, subject characteristics, participants' ages and genders, methods of MMP-2 measurement, and MMP-2 levels in atherosclerotic CAD into a table.

### 2.4. Risk of Bias Assessment

The Newcastle–Ottawa Scale (NOS) which has three domains for both case–control and cohort studies was used by two reviewers (NS and AA) to assess the quality of risk of bias [32]. For case–control studies, NOS evaluated the choice of study groups (including cases and controls), the comparability of the groups, and the determination of exposure for both the cases and the control groups. In contrast, for cohort study, NOS evaluated the choice of study groups (exposed and nonexposed), comparability across groups, and evaluation of outcome. There were eight items in each of the three domains that may be given a star rating. A minimum of one star or a maximum of two stars were assigned to each item. Studies with seven to nine stars were deemed to be of high-quality studies, while those with four to six stars were deemed to be of fair quality studies and those with one to three stars to be of low-quality studies.

## 3. Results

**Figure 2:**
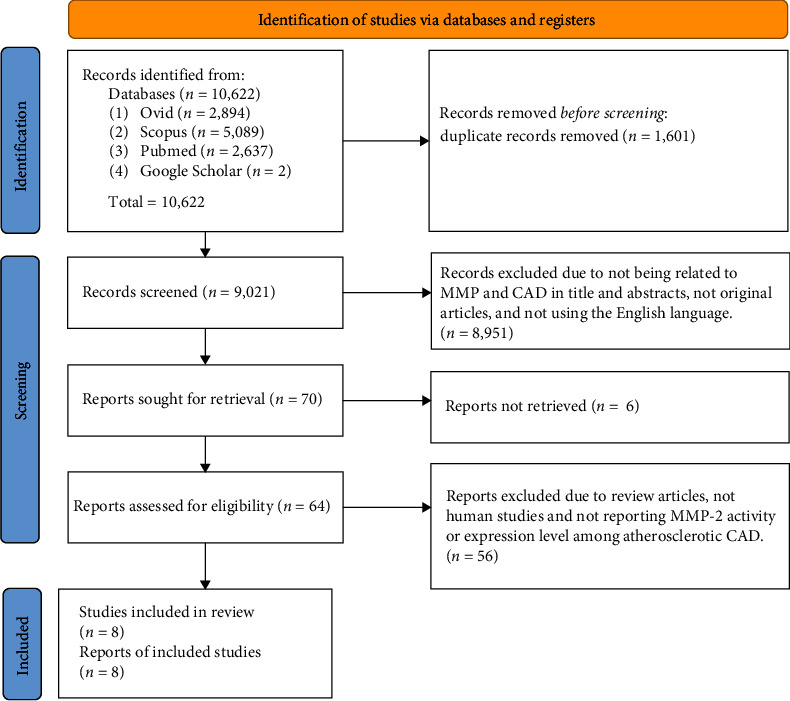
Flow chart of the article selection.

A total of 10,622 articles were obtained from four online databases: Ovid (2,894), Scopus (5,089), PubMed (2,637), and Google Scholar (2). The articles were published between 2018 and February 2023. Then, 1,601 articles were excluded due to replication. Following the assessment of the title and abstract of the articles, 8,951 articles were further excluded. The remaining 64 articles' complete texts were attained and thoroughly assessed. From these 64 articles, only eight were selected to be included in this study. The 56 excluded articles were listed with their respective exclusion reason in Table 3.  Figure 2 displays the article selection process.

Table 4 summarized the details of all the final eight studies. Four studies were case–control clinical trials, three studies were cross-sectional studies and one study was a cohort study by design. Quality assessment using NOS revealed that the score range of the studies reviewed was between 4 and 9 (fair to high quality). All the subjects were patients diagnosed with CAD. The subject's age ranged from 18 to 79 years old. Most of the studies measured MMP-2 levels in the serum [33–35], plasma [38, 40], and genomic DNA isolated from the subject's blood [39]. Additionally, two studies also investigated MMP-2 expression in the coronary atherosclerotic plaque [36, 37].

A potent natural anti-inflammatory agent, curcumin was found to significantly inhibit the MMP-2 activity in the serum of CAD patients in comparison to the placebo group (*p* < 0.001) [33]. In addition, Sai et al. [34] demonstrated a higher MMP-2 levels in the serum of AMI patients compared with the healthy subjects. There was also a significantly lower serum MMP-2 levels in AMI patients posttreatment with benazepril and rosuvastatin, compared with those who only received rosuvastatin (*p* < 0.05) [34].

Moreover, Li et al. [35] showed a significantly higher MMP-2 serum levels in patients with unstable CAD than in stable CAD and healthy groups. Murashov et al. [37] also demonstrated a higher expression of MMP-2 by 7.8 times in unstable atherosclerotic plaques compared with stable atherosclerotic plaques via immunohistochemistry (IHC) analysis (*p* < 0.05). MMP was mostly expressed in the cytoplasm of foamy macrophages in atheromatous core and caps of unstable plaques [36]. However, MMP-2 expression did not significantly differ between the three types of unstable atherosclerotic plaque (degenerative-necrotic type, lipid type, and inflammatory-erosive type) [37]. A study by Owolabi et al. [40] found no significant difference in MMP-2 levels between the AMI and stable CAD groups at any time point.

Besides, Melin et al. [38] found that plasma MMP-2 levels were higher in Type 1 diabetic patients with high-MMP-14 levels. High-MMP-14 levels was associated with increased CVD in this group of patients [38]. Malkani et al. [39] found two MMP-2 gene single nucleotide polymorphisms (SNPs); namely rs243865 and rs243866 in patients with atherosclerosis. The CA, CG, and TA haplotypes of the MMP-2 gene were significantly connected with atherosclerosis [39].

## 4. Discussion

The aim of this review was to determine the role of MMP-2 in atherosclerosis development in CAD patients. MMP-2 levels were discovered to be higher in CAD patients than in healthy individuals. Meanwhile, amongst patients with CAD, MMP-2 levels were higher in unstable than in stable atherosclerotic plaque, indicating that MMP-2 plays a role in the vulnerability and severity of the atherosclerotic plaque. However, one study did not support such findings, in which no significant difference in MMP-2 levels was found in acute MI and stable CAD [40]. This might be because of the nature of the study that examined MMP-2 levels following cardiac catheterization, which is a procedure to clear the blockage that might disturb the MMP-2 levels. MMP-2 was also found to be related to high levels of MMP-14. MMP-14 is a pro-MMP-2 activator that is linked to the incidence of CVD. Two SNPs of the MMP-2 gene (rs243865 and rs243866) were associated with atherosclerosis. Moreover, treatment of CAD patients with ACE inhibitor, lipid-lowering drug, and anti-inflammatory compound decreased MMP-2 activity and levels, indicating that the inhibition of MMP-2 is useful in treating CAD.

Atherosclerosis is a chronic inflammatory disease involving the deposition of cholesterol, fats, blood cells, and other substances that form a plaque inside the vessel wall [41]. It is the main underlying cause of CAD, in which the atherosclerotic plaque buildup causes narrowing of the arterial lumen and disrupts the blood supply to the heart [42]. In the worst-case scenario, the atherosclerotic plaque could rupture, leading to thrombus formation and complete occlusion of the coronary arteries that presents as ACS [13].

MMPs participate in various stages of atherosclerosis development, from lesion initiation to plaque rupture. MMP-2 is widely distributed in endothelial cells, VSMCs, leukocytes, platelets, adventitia, and dermal fibroblasts [14], and is essential for the development of atherosclerosis [15–18, 43, 44]. Atherosclerosis starts with endothelial dysfunction that promotes infiltration of low-density lipoprotein (LDL) into the intima, which becomes oxidized to form oxidized LDL (oxLDL). MMP-2 promotes endothelial dysfunction by causing proteolytic degradation of endothelial nitric oxide synthase (eNOS) and its cofactor, heat shock protein 90 (HSP90). This leads to reduced endothelial nitric oxide (NO) production [15]. Incubating bovine coronary artery endothelial cells with recombinant MMP-2 significantly decreased NO release. Furthermore, MMP-2 cleaves HSP90 into several fragments, thus disrupting eNOS activity [15].

Subsequently, the macrophages take up oxLDL in the intima to form foam cells. Circulating and activated platelets facilitate this stage of atherosclerosis formation. MMP-2 expressed by the circulating platelets is higher in patients with CAD compared with healthy people [16]. A study by Cheung et al. [45] found the increased release of MMP-2 from platelets activated by oxidative stress in acutely ill neonates. MMP-2 expressed by the activated platelets has an important role in facilitating monocyte infiltration into the intima to become macrophages [16]. This platelet MMP-2 interacts with endothelial cell's protease-activated receptor 1 (PAR-1) and increases vascular cell adhesion molecule 1 (VCAM-1) expression. VCAM-1 encourages monocyte adherence and infiltration via endothelial cells into the subintimal layer, which contributes to the development of atherosclerosis [16, 17].

OxLDL is also responsible for the migration of VSMCs into the intima. The VSMCs migrate from media into the intima, forming fibrous cap of the atherosclerosis plaque [46]. The sphingomyelin/ceramide/sphingosine-1-phosphate (Spm/Cer/S1P) pathway is one of the signaling pathways involved in VSMC proliferation induced by oxLDL [18]. By activating this pathway, MMP-2 stimulates oxLDL-induced VSMCs proliferation in atherosclerosis [18]. It is also supported by the silencing of MMP-2 gene inhibits the proliferation of oxLDL-induced VSMCs [18].

MMPs are also participate in modulating the progression of stable atherosclerotic plaque to unstable plaque by degrading the ECM [47, 48]. Degradation of ECM causes thinning of the plaque fibrous cap, and facilitates the plaque to become unstable and prone to rupture [49, 50]. An unstable atherosclerotic plaque is responsible for the life-threatening clinical conditions such as AMI [51]. Previous study showed that 4-Hydroxynonenal (HNE), a highly reactive product of lipid peroxidation enhanced MMP-2 production in the fibrous cap's VSMCs by activating the activating tyrosine kinase/Nuclear factor kappa beta (Akt/NF-ĸ*β*) signaling pathways [43]. There was an increase in MMP-2 mRNA and protein expression in VSMCs following exposure to HNE, indicating that HNE regulates MMP-2 production at transcriptional level [43]. Given that HNE is consistently present in a significant amount in the unstable atherosclerotic plaque, it is postulated that HNE accelerates plaque rupture through enhanced production of MMP-2 [52]. This is supported by previous studies that demonstrate a higher MMP-2 expression and activity in vulnerable regions of the atherosclerotic plaque [53, 54].

**Figure 3:**
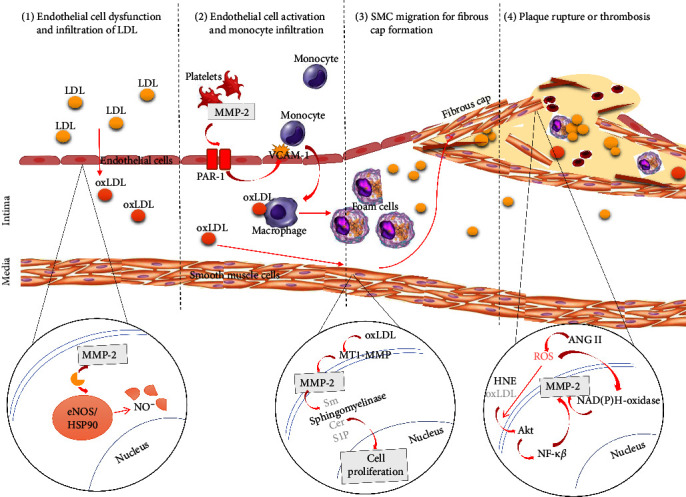
Signaling pathways of MMP-2 in atherosclerosis development. Initially, MMP-2 cleaves eNOS or its cofactor, HSP90 to cause endothelial dysfunction and facilitate infiltration of LDL into the intima. Then, MMP-2 expressed by activated platelets promote monocyte transmigration into the intima through endothelial PAR-1 activation. MMP-2 also facilitates oxLDL-induced VSMCs migration to the intima, forming fibrous cap in the atherosclerotic plaque. Last, MMP-2 stimulates fibrous cap degradation and rupture of atherosclerotic plaque via several pathways including Akt-NF-ĸ*β* pathway which is induced by HNE, and NADPH oxidase pathway which is induced by ANG II and ROS. Akt = activating tyrosine kinase, ANG II, angiotensin II; eNOS, endothelial nitric oxide synthase; HSP90, heat shock protein 90; HNE, hydroxynoneal; LDL, low-density lipoprotein; MMP-2, matrix metalloproteinase-2; MT1-MM*P*, membrane Type1-matrix metalloproteinase; NAD(P)H, nicotinamide adenine dinucleotide phosphate; NO, nitric oxide; NF-ĸ*β*, nuclear factor kappa beta; oxLDL, oxidized low density lipoprotein; PAR-1, protease-activated receptor 1; ROS, reactive oxygen species; Sm/Cer/S1P, sphingomyelin/ceramide/sphingosine-1-phosphate; VCAM-1, vascular cell adhesion molecule 1.

Furthermore, a vasoactive peptide, angiotensin II (ANG II) has been found to enhance MMP-2 mRNA expression in the fibrous cap's VSMCs by activating NADPH oxidase in a p47phox-dependent manner [44]. VSMCs isolated from wild-type (WT) mice showed a significant increase in MMP-2 mRNA expression following exposure to ANG II. In contrast, VSMCs isolated from p47phox−/− mice secreted less MMP-2 compared with the VSMCs of WT mice. This indicates that ANG II-stimulated MMP-2 expression is dependent on the p47phox subunit of NADPH-oxidase. Besides, MMP-2, p47phox and ANG II were colocalized in VSMCs-rich areas of atherosclerotic plaques, suggesting their functional interactions [55]. Furthermore, ANG II stimulates reactive oxygen species (ROS) production that alters the plaque proteolytic balance by enhancing VSMC's NADPH oxidase-dependent release of MMP-2. This eventually leads to plaque instability [44]. The relevant signaling pathways related to MMP-2 and the various stages of atherosclerosis development are summarized in Figure 3.

## 5. Study Limitation

A few limitations have been identified from this review. First, there is only one study that focuses on association of MMP-2 gene polymorphisms with the occurrence of atherosclerosis and CAD. Hence, more studies on MMP-2 gene polymorphisms in association with CAD are needed. Second, there is a limited data on MMP-2 signaling pathways that is based on human study. Most of the data on the signaling pathways were derived from *in vitro* and *in vivo* preclinical studies. Therefore, further studies related to MMP-2 signaling pathways using human samples are needed in the future.

## 6. Conclusion

To conclude, this review has identified some studies that demonstrate the role of MMP-2 in atherosclerosis progression among patients with CAD. MMP-2 expression and activity are found to be higher in patients with CAD. Besides, MMP-2 participates in various stages of atherosclerotic plaque development, which is the main pathophysiology underlying CAD. This review could increase our understanding on the role of MMPs, specifically MMP-2 in atherosclerosis development among CAD patients, which could help in early disease diagnosis and development of targeted therapy via the MMP-2 signaling pathway.

## Figures and Tables

**Table 1:** PRISMA 2020 item checklist [31].

Section and topic	Item #	Checklist item	Location where item is reported
Title	1	Identify the report as a systematic review	Title, line 2
Abstract	2	See the PRISMA 2020 for Abstracts checklist	Table 2
Introduction			
Rationale	3	Describe the rationale for the review in the context of existing knowledge.	Introduction, paragraphs 1–6
Objectives	4	Provide an explicit statement of the objective(s) or question(s) the review addresses	Introduction, paragraph 6, line 10
Methods			
Eligibility criteria	5	Specify the inclusion and exclusion criteria for the review and how studies were grouped for the syntheses	Methodology, see 2.2
Information sources	6	Specify all databases, registers, websites, organizations, reference lists, and other sources searched or consulted to identify studies. Specify the date when each source was last searched or consulted	Methodology, see 2.1
Search strategy	7	Present the full search strategies for all databases, registers and websites, including any filters and limits used	Methodology, see 2.1
Selection process	8	Specify the methods used to decide whether a study met the inclusion criteria of the review, including how many reviewers screened each record and each report retrieved, whether they worked independently, and if applicable, details of automation tools used in the process	Methodology, see 2.3
Data collection process	9	Specify the methods used to collect data from reports, including how many reviewers collected data from each report, whether they worked independently, any processes for obtaining or confirming data from study investigators, and if applicable, details or automation tools used in the process	Methodology, see 2.3
Data items	10a	List and define all outcomes for which data were sought. Specify whether all results that were compatible with each outcome domain in each study were sought (e.g., for all measures, time points, analyses), and if not, the methods used to decide which results to collect	Methodology, see 2.3
	10b	List and define all other variables for which data were sought (e.g., participant and intervention characteristics, funding sources). Describe any assumptions made about any missing or unclear information	Table 5
Study risk of bias assessment	11	Specify the methods used to assess risk of bias in the included studies, including details of the tool(s) used, how many reviewers assessed each study and whether they worked independently, and if applicable, details of automation tools used in the process	Methodology, see 2.4
Effect measures	12	Specify for each outcome the effect measure(s) (e.g., risk ratio, mean difference) used in the synthesis or presentation of results	Methodology, see 2.3, line 6.
Synthesis methods	13a	Describe the processes used to decide which studies were eligible for each synthesis (e.g., tabulating the study intervention characteristics and comparing against the planned groups for each synthesis (item #5))	Methodology, see 2.3, line 4–6.
	13b	Describe any methods required to prepare the data for presentation or synthesis, such as handling of missing summary statistics, or data conversions	Involving article exporting from database into mendeley web taught by Mrs. Norizam Salamat
	13c	Describe any methods used to tabulate or visually display results of individual studies and syntheses.	Not applicable
	13d	Describe any methods used to synthesize results and provide a rationale for the choice(s). If meta-analysis was performed, describe the model(s), method(s) to identify the presence and extent of statistical heterogeneity, and software package(s) used	No statistical analysis was done
	13e	Describe any methods used to explore possible causes of heterogeneity among study results (e.g., subgroup analysis, metaregression).	Not applicable
	13f	Describe any sensitivity analyses conducted to assess robustness of the synthesized results	Not applicable
Reporting bias assessment	14	Describe any methods used to assess risk of bias due to missing results in a synthesis (arising from reporting biases).	Not applicable
Certainty assessment	15	Describe any methods used to assess certainty (or confidence) in the body of evidence for an outcome	Not applicable
Results			
Study selection	16a	Describe the results of the search and selection process, from the number of records identified in the search to the number of studies included in the review, ideally using a flow diagram (see Figure 1)	Results, paragraph 1 and see Figure 2
	16b	Cite studies that might appear to meet the inclusion criteria, but which were excluded, and explain why they were excluded	Table 3
Study characteristics	17	Cite each included study and present its characteristics.	Results, paragraph 2–5 and see Table 4
Risk of bias in studies	18	Present assessments of risk of bias for each included study	Results, paragraph 2, line 3, and see Table 4, column 6
Results of individual studies	19	For all outcomes, present, for each study: (a) summary statistics for each group (where appropriate) and (b) an effect estimate and its precision (e.g., confidence/credible interval), ideally using structured tables or plots	Results, see Table 4, column 5
Results of syntheses	20a	For each synthesis, in brief summarize the characteristics and risk of bias among contributing studies	Results, see Table 4, column 2 and 6
	20b	Present results of all statistical syntheses conducted. If meta-analysis was done, present for each the summary estimate and its precision (e.g., confidence/credible interval) and measures of statistical heterogeneity. If comparing groups, describe the direction of the effect	Results, see Table 4, column 5
	20c	Present results of all investigations of possible causes of heterogeneity among study results	Not applicable
	20d	Present results of all sensitivity analyses conducted to assess the robustness of the synthesized results	Not applicable
Reporting biases	21	Present assessments of risk of bias due to missing results (arising from reporting biases) for each synthesis assessed	Not applicable
Certainty of evidence	22	Present assessments of certainty (or confidence) in the body of evidence for each outcome assessed	Not applicable
Discussion	23a	Provide a general interpretation of the results in the context of other evidence	Discussion, paragraph 1–7
	23b	Discuss any limitations of the evidence included in the review	Not applicable
	23c	Discuss any limitations of the review processes used	Study limitation
	23d	Discuss implications of the results for practice, policy, and future research	Conclusion
Other information			
Registration and protocol	24a	Provide registration information for the review, including register name and registration number, or state that the review was not registered	Protocol registration
	24b	Indicate where the review protocol can be accessed, or state that a protocol was not prepared	Protocol registration
	24c	Describe and explain any amendments to information provided at registration or in the protocol.	Not applicable
Support	25	Describe sources of financial or nonfinancial support for the review, and the role of the funders or sponsors in the review	Acknowledgement
Competing interests	26	Declare any competing interests of review authors	Conflict of interest
Availability of data, code, and other materials	27	Report which of the following are publicly available and where they can be found: template data collection forms; data extracted from included studies; data used for all analyses; analytic code; any other materials used in the review	Data availability statement

**Table 2:** PRISMA 2020 for abstract checklist [31].

Section and topic	Item #	Checklist item	Location where Item is reported
Title	1	Identify the report as a systematic review	Abstract, line 5.
Background			
Objectives	2	Provide an explicit statement of the main objective (s) or question (s) the review addresses	Abstract, line 5
Methods			
Eligibility criteria	3	Specify the inclusion and exclusion criteria for the review	Not stated in abstract
Information sources	4	Specify the information sources (e.g., databases and registers) used to identify studies and the date when each was last searched	Abstract, line 6 and 7
Risk of bias	5	Specify the methods used to assess risk of bias in the included studies	Abstract, line 7
Synthesis of results	6	Specify the methods used to present and synthesize results	Abstract, line 6–8
Results			
Included studies	7	Give the total number of included studies and participants and summarize relevant characteristics of studies	Abstract, line 8
Synthesis of results	8	Present results for main outcomes, preferably indicating the number of included studies and participants for each. If meta-analysis was done, reports the summary estimate and confidence/credible interval. If comparing groups, indicate the direction of the effects (i.e., which group is favored)	Abstract, line 8–12
Discussion			
Limitation of evidence	9	Provide a brief summary of the limitations of the evidence included in the review (e.g., study risk of bias, inconsistency, and imprecision).	Not stated in abstracts
Interpretation	10	Provide a general interpretation of the results and important implications	Abstract, line 13 and 14
Other			
Funding	11	Specify the primary source of funding for the review	Not stated in abstracts
Registration	12	Provide the register name and registration number	Not stated in abstracts

**Table 3:** List of references and their exclusion reason.

	References	Exclusion reason
1	A. Ben Braiek, H. Chahed, F. Dumont, F. Abdelhak, D. Hichem, H. Gamra, and B. Baudin, “Identification of biomarker panels as predictors of severity in coronary artery disease,” *Journal of Cellular and Molecular Medicine*, vol. 25(3), pp. 1518-1530, 2021	Not reporting MMP-2
2	A. A. Elhewala, M. Sanad, A. M. Soliman, M. A. Y. M. Sami, and A. A. Ahmed, “Matrix metalloproteinase-9 in pediatric rheumatic heart disease with and without heart failure,” *Biomedical Reports*, vol. 14(1), pp. 4, 2021	Not reporting MMP-2
3	A. Gugerell, K. Zlabinger, D. Lukovic, J. Winkler, R. Hemetsberger, L. Mandic, D. Traxler, A. Spannbauer, N. Pavo, and M. Gyongyosi, “Effect of MMP-2 on compromised homing of intracoronary delivery of mesenchymal stem cell in a porcine reperfused myocardial infarction: comparison with intramyocardial cell delivery,” *Cardiovascular Research*, vol. 114 (1), pp. S28–S29, 2018.	An *in vitro* study
4	A. J. Mouton, O. J. Rivera Gonzalez, A. R. Kaminski, E. T. Moore, and M. L. Lindsey, “Matrix metalloproteinase-12 as an endogenous resolution promoting factor following myocardial infarction,” *Pharmacological Research*, vol. 137, pp. 252-258, 2018.	A review article and not reporting MMP-2
5	A. Kubota, A. Suto, K. Suzuki, Y. Kobayashi, and H. Nakajima, “Matrix metalloproteinase-12 produced by Ly6Clow macrophages prolongs the survival after myocardial infarction by preventing neutrophil influx,” *Journal of Molecular and Cellular Cardiology*, vol. 131, pp. 41–52, 2019.	Not reporting MMP-2
6	A. Shevchenko, V. Prokofiev, V. Konenkov, and Y. Ragino, “Association of inflammation, destruction and angiogenesis genes polymorphism with serum levels of matrix metalloproteinases 3 and 9 in patients with coronary atherosclerosis,” *Ural-Siberian Conference on Computational Technologies in Cognitive Science*, *Genomics and Biomedicine* (*CSGB*), *Novosibirsk*, *Russian Federation*, pp. 290-294, 2022.	Not reporting MMP-2
7	C. A. Meschiari, M. Jung, R. P. Iyer, A. Yabluchanskiy, H. Toba, M. R. Garrett, and M. L. Lindsey, “Macrophage overexpression of matrix metalloproteinase-9 in aged mice improves diastolic physiology and cardiac wound healing after myocardial infarction. American Journal of Physiology,” *Heart and Circulatory Physiology*, vol. 314(2), pp. H224–H235, 2018.	Not reporting MMP-2
8	C. Guan, Y. Xiao, K. Li, T. Wang, Y. Liang, and G. Liao, “MMP-12 regulates proliferation of mouse macrophages via the ERK/P38 MAPK pathways during inflammation,” *Experimental Cell Research*, vol. 378(2), pp. 182-190, 2019.	An *in vivo* study and not reporting MMP-2
9	C. Wang, C. Song, Q. Liu, R. Zhang, R. Fu, H. Wang, D. Yin, W. Song, H. Zhang, and K. Dou, “Gene Expression Analysis Suggests Immunological Changes of Peripheral Blood Monocytes in the Progression of Patients With Coronary Artery Disease,” *Frontiers in Genetics*, vol.12, pp. 641117, 2021.	Not reporting MMP-2
10	D. Balakrishna, B. Sowjanya, M. Prasad, and R. Viswakumar, “Age- And Gender-Based Predisposition Of MMP-9 -1562 C > T Genotype And Allele Frequencies With Serum MMP-9 Levels As Probable Risk Factors In Patients With Coronary Artery Disease,” *Indian Journal of Clinical Biochemistry*, 2022.	Not reporting MMP-2
11	D. J. Medina-Leyte, O. Zepeda-García, M. Domínguez-Pérez, A. González-Garrido, T. Villarreal-Molina, and L. Jacobo-Albavera, “Endothelial Dysfunction, Inflammation and Coronary Artery Disease: Potential Biomarkers and Promising Therapeutical Approaches,” *International Journal of Molecular Sciences*, vol. 22(8), pp. 3850, 2021.	A review article
12	D. Opincariu, I. Rodean, N. Rat, R. Hodas, I. Benedek, and T. Benedek, “Systemic Vulnerability, as Expressed by I-CAM and MMP-9 at Presentation, Predicts One Year Outcomes in Patients with Acute Myocardial Infarction-Insights from the VIP Clinical Study,” *Journal of Clinical Medicine*, vol. 10(15), pp. 3435, 2021.	Not reporting MMP-2
13	D. Wang, X. Lou, X. M. Jiang, C. Yang, X. L. Liu, and N. Zhang, “Quercetin protects against inflammation, MMP-2 activation and apoptosis induction in rat model of cardiopulmonary resuscitation through modulating Bmi-1 expression,” *Molecular Medicine Reports*, vol. 18(1), pp. 610-616, 2018.	An *in vivo* study
14	H. Funayama, T. Yoshioka, S. E. Ishikawa, S. I. Momomura, and K. Kario, “Close Association of Matrix Metalloproteinase-9 Levels With the Presence of Thin-Cap Fibroatheroma in Acute Coronary Syndrome Patients: Assessment by Optical Coherence Tomography and Intravascular Ultrasonography,” *Cardiovascular Revascularization Medicine*, vol. 32, pp. 5-10, 2021.	Not reporting MMP-2
15	G. Angelini, D. Flego, R. Vinci, D. Pedicino, F. Trotta, A. Ruggio, G. P. Piemontese, D. Galante, M. Ponzo, L. M. Biasucci, G. Liuzzo, and F. Crea, “Matrix metalloproteinase-9 might affect adaptive immunity in non-ST segment elevation acute coronary syndromes by increasing CD31 cleavage on CD4+ T-cells,” *European Heart Journal*, vol. 39(13), pp. 1089-1097, 2018	Not reporting MMP-2
16	G. Kremastiotis, I. Handa, C. Jackson, S. George, and J. Johnson, “Disparate effects of MMP and TIMP modulation on coronary atherosclerosis and associated myocardial fibrosis,” *Scientific Reports*, vol. 11(1), pp. 23081, 2021.	Not reporting MMP-2
17	G. Sangeethadevi, S. U. V V, R. A. R. Jansy Isabella, G. Saravanan, P. Ponmurugan, P. Chandrasekaran, S. Sengottuvelu, and S. Vadivukkarasi, “Attenuation of lipid metabolic abnormalities, proinflammatory cytokines, and matrix metalloproteinase expression by biochanin-A in isoproterenol-induced myocardial infarction in rats,” *Drug and Chemical Toxicology*, vol. 45(5), pp. 1951-1962, 2022.	An *in vivo* study
18	G. Simões, T. Pereira, A. Caseiro, G. Simoes, T. Pereira, and A. Caseiro, “Matrix metalloproteinases in vascular pathology,” *Microvascular Research*, vol. 143, pp. 104398, 2022.	A review article
19	H. F. Ebrahim, F. F. Abdel Hamid, M. A. Haykal, and A. F. Soliman, “Cyclophilin A and matrix metalloproteinase-9: Their relationship, association with, and diagnostic relevance in stable coronary artery disease”, *Vascular*, vol. 28(2), pp. 212-221, 2020.	Not reporting MMP-2
20	I. Guizani, W. Zidi, Y. Zayani, F. Nesrine, H. Douik, H. Sanhaji, M. S, Mourali, M. Feki, and M. Allal-Elasmi, “Matrix metalloproteinase 3 and 9 as genetic biomarkers for the occurrence of cardiovascular complications in coronary artery disease: a prospective cohort study,” *Molecular Biology Reports*, vol. 49(10), pp. 9171-9179, 2022.	Not reporting MMP-2
21	J. M. Howes, N. Pugh, S. W. Hamaia, S. M. Jung, V. Knäuper, J. D. Malcor, R. W. Farndale, “MMP-13 binds to platelet receptors *α*IIb*β*3 and GPVI and impairs aggregation and thrombus formation,” *Research & Practice in Thrombosis & Haemostasis*, vol. 2(2), pp. 370-379, 2018.	Not reporting MMP-2
22	J. Nordeng, H. Schandiz, S. Solheim, S. Åkra, P. Hoffman, B. Roald, B. Bendz, H. Arnesen, R. Helseth, and I. Seljeflot, “TIMP-1 expression in coronary thrombi associate with myocardial injury in ST-elevation myocardial infarction patients. *Coronary Artery Disease*, vol. 33(6), pp. 446-455, 2022.	Not reporting MMP-2
23	K. Brassington, S. Selemidis, S. Bozinovski, and R. Vlahos, “Chronic obstructive pulmonary disease and atherosclerosis: common mechanisms and novel therapeutics,” *Clinical Science*, vol. 136(6), pp. 405-423, 2022.	A review article
24	K. Pahk, C. Joung, H. Y. Song, S. Kim, and W. Kim, “A Novel Cd147 Inhibitor Sp-8356 Attenuates Plaque Progression And Stabilizes Vulnerable Plaque In Apoe-Deficient Mice,” *Journal of Hypertension*, vol. 37, pp. e105–e106, 2019.	An *in vivo* study
25	L. Li, J. Li, J. Yi, H. Liu, and H. Lei, “Dose-Effect of Irbesartan on Cyclooxygenase-2 and Matrix Metalloproteinase-9 Expression in Rabbit Atherosclerosis,” *Journal of Cardiovascular Pharmacology*, vol. 71(2), pp. 82-94, 2018.	An *in vivo* study and not reporting MMP-2
26	L. Yang, H. G. Sui, M. M, Wang, J. Y. Li, X. F. He, J. Y. Li, and X. Z. Wang, “MiR-30c-1-3p targets matrix metalloproteinase 9 involved in the rupture of abdominal aortic aneurysms,” *Journal of Molecular Medicine*, vol. 100(8), pp. 1209-1221, 2022.	Not reporting MMP-2
27	L. Schirone, M. Forte, L. D'Ambrosio, V. Valenti, D. Vecchio, S. Schiavon, G. Spinosa, G. Sarto, V. Petrozza, G. Frati, and S. Sciarretta, “An Overview of the Molecular Mechanisms Associated with Myocardial Ischemic Injury: State of the Art and Translational Perspectives,” *Cells*, vol. 11(7), pp. 1165, 2022.	A review article
28	M. K. Georgakis, S. W. Van Der Laan, Y. Asare, J. M. Mekke, S, Haitjema, A. H. Schoneveld, S. C. A. De Jager, N. S. Nurmohamed, J. Kroon, E. S. G. Stroes, D. P. V. De Kleijn, G. J. De Borst, L. Maegdefessel, O. Soehnlein, G. Pasterkamp, and M. Dichgans, “Monocyte-Chemoattractant Protein-1 Levels in Human Atherosclerotic Lesions Associate with Plaque Vulnerability,” *Arteriosclerosis*, *Thrombosis*, *and Vascular Biology*, vol. 41 (6), pp. 2038-2048, 2021.	Not reporting relation with MMP-2 and/or CAD
29	M. Medhet, W. El-Bakly, A. Badr, A. Awad, and E. El-Demerdash, “Thymoquinone attenuates isoproterenol-induced myocardial infarction by inhibiting cytochrome C and matrix metalloproteinase-9 expression,” *Clinical and Experimental Pharmacology and Physiology*, vol. 49 (3), pp. 391-405, 2022.	Not reporting MMP-2
30	M. Kumric, J. A. Borovac, D. Martinovic, T. Ticinovic Kurir, and J. Bozic, “Circulating Biomarkers Reflecting Destabilization Mechanisms of Coronary Artery Plaques: Are We Looking for the Impossible?,” *Biomolecules*, vol. 11(6), pp. 881, 2021.	A review article
31	M. Sabry, S. Mostafa, L. Rashed, M. Abdelgwad, S. Kamar, and S. Estaphan, “Matrix metalloproteinase 9 a potential major player connecting atherosclerosis and osteoporosis in high fat diet fed rats,” *PLOS ONE*, vol. 16(2), 2021.	An *in vivo* study
32	M. Sheikhvatan, M. A. Boroumand, M. Behmanesh, and S. Ziaee, “Association of R279Q and C1562T polymorphisms of matrix metalloproteinase 9 gene and increased risk for myocardial infarction in patients with premature coronary artery disease,” *Journal of Clinical Laboratory Analysis*, vol. 32(1), 2018.	Not reporting MMP-2
33	M. Skrzypiec-Spring, J. Urbaniak, A. Sapa-Wojciechowska, A. Pietkiewicz, A. Orda, B. Karolko, R. Danielewicz, I. Bil-Lula, M. Woźniak, R. Schulz, and A. Szeląg, “Matrix Metalloproteinase-2 Inhibition in Acute Ischemia-Reperfusion Heart Injury-Cardioprotective Properties of Carvedilol,” *Pharmaceuticals* (*Basel*), vo. 14(12), pp. 1276, 2021.	An *in vivo* study
34	M. U. Somuncu, H. Pusuroglu, H. Karakurt, I. I. Bolat, S. T. Karakurt, A. R. Demir, N. Isiksacan, O. Akgul, O. Surgit, N. Isıksacan, O. Akgul, and O. Surgit, “The prognostic value of elevated matrix metalloproteinase-9 in patients undergoing primary percutaneous coronary intervention for ST-elevation myocardial infarction: A two-year prospective study,” *Revista Portuguesa de Cardiologia*, vol. 39(5), pp. 267–276, 2020.	Not reporting MMP-2
35	M. M Zhang, X. W. Chang, X. Q. Hao, H. Wang, X. Xie, and S. Y. Zhang, “Association between matrix metalloproteinase 9 C-1562T polymorphism and the risk of coronary artery disease: an update systematic review and meta-analysis,” *Oncotarget*, vol. 9(10), pp. 9468–9479, 2018.	Not reporting MMP-2
36	N. Malkani, M. Farheen, H. Hamid, A. Batool, R. U. Khan, and A. Yaqub, “Matrix metalloprotease-9 polymorphism and its association with Atherosclerosis—A case-control study in Pakistani population,” *Journal of Pakistan Medical Association*, vol. 69(10), pp. 1416-1420, 2019.	Not reporting MMP-2
37	P. R. Gonçalves, L. D. Nascimento, R. F. Gerlach, K. E. Rodrigues, and A. F. Prado, “Matrix Metalloproteinase 2 as a Pharmacological Target in Heart Failure,” *Pharmaceuticals* (*Basel*), vol. 15(8), pp. 920, 2022.	A review article
38	P. Libby, J. E. Buring, L. Badimon, G. K. Hansson, J. Deanfield, M. S. Bittencourt, L. Tokgözoğlu, and E. F. Lewis, “Atherosclerosis,” *Nature Reviews Disease Primers*, vol. 5(1), 2019.	A review article
39	P. Theofilis, M. Sagris, A. S. Antonopoulos, E. Oikonomou, K. Tsioufis, and D. Tousoulis, “Non-Invasive Modalities in the Assessment of Vulnerable Coronary Atherosclerotic Plaques,” *Tomography*, vol. 8(4), pp.1742-1758, 2022.	A review article
40	R. Hassanzadeh-Makoui, B. Razi, S. Aslani, D. Imani, and S. S. Tabaee, “The association between Matrix Metallo-proteinases-9 (MMP-9) gene family polymorphisms and risk of Coronary Artery Disease (CAD): a systematic review and meta-analysis,” *BMC Cardiovascular Disorders*, vol. 20(1), pp. 232, 2020.	A systematic review and meta-analysis article
41	R. Huang, Y. Xin, R. Zhu, J. Song, M. Luo, and D. A. Chen, “Meta-Analysis of Matrix Metalloproteinases in the Risk of Cardiovascular and Neurodegenerative Diseases,” *BioMed Research International*, vol. 2022, pp. 3360316, 2022.	A systematic review and meta-analysis article
42	S. Iwańczyk, T. Lehmann, M. Grygier, P. Woźniak, M. Lesiak, and A. Araszkiewicz, “Serum matrix metalloproteinase-8 level in patients with coronary artery abnormal dilatation,” *Polish Archives of Internal Medicine*, vol. 132(5), pp. 16241, 2022.	Not reporting MMP-2
43	S. J. George, and J. L. Johnson, “Investigation of Atherosclerotic Plaque Vulnerability,” *Methods in Molecular Biology*, vol. 2419, pp. 521-535, 2022.	Book chapters
44	S. Jebari-Benslaiman, U. Galicia-García, A. Larrea-Sebal, J. R. Olaetxea, I. Alloza, K. Vandenbroeck, A. Benito-Vicente, and C. Martín, “Pathophysiology of Atherosclerosis,” *International Journal of Molecular Sciences*, vol. 23(6), pp. 3346, 2022.	Not reporting relation with MMP-2 and/or CAD
45	S. Shi, and J. L.Yi, “S100A8/A9 promotes MMP-9 expression in the fibroblasts from cardiac rupture after myocardial infarction by inducing macrophages secreting TNF*α*,” *European Review for Medical and Pharmacological Sciences*, vol. 22(12), pp. 3925-3935, 2018.	Not reporting MMP-2
46	S. Zhang, Y. Liu, Y. Cao, S. Zhang, J. Sun, Y. Wang, S. Song, H. Zhang, “Targeting the Microenvironment of Vulnerable Atherosclerotic Plaques: An Emerging Diagnosis and Therapy Strategy for Atherosclerosis,” *Advanced Materials*, vol. 34 (29), 2022.	A review article
47	T. Radhiga, S. Senthil, A. Sundaresan, and K. V. Pugalendi. “Ursolic acid modulates MMPs, collagen-I, *α*-SMA, and TGF-*β* expression in isoproterenol-induced myocardial infarction in rats,” *Human & Experimental Toxicology*, vol. 38(7):785-793, 2019.	An *in vivo* study
48	V. Lubrano, and S. Balzan, “Status of biomarkers for the identification of stable or vulnerable plaques in atherosclerosis,” *Clinical Science*, vol. 135(16), pp. 1981-1997, 2021.	A review article
49	W. Gong, Y. Ma, A. Li, H. Shi, and S. Nie, “Trimetazidine suppresses oxidative stress, inhibits MMP-2 and MMP-9 expression, and prevents cardiac rupture in mice with myocardial infarction,” *Cardiovascular Therapeutics*, vol. 36(5), pp. e12460, 2018.	An *in vivo* study
50	W. Hu, R. Wei, L. Wang, J. Lu, H. Liu, and W. Zhang, “Correlations of MMP-1, MMP-3, and MMP-12 with the degree of atherosclerosis, plaque stability and cardiovascular and cerebrovascular events,” *Experimental and Therapeutic Medicine*, vol. 15(2), pp. 1994-1998, 2018.	Not reporting MMP-2
51	W. Wu, D. Liu, S. Jiang, K. Zhang, H. Zhou, and Q. Lu. “Polymorphisms in gene MMP-2 modify the association of cadmium exposure with hypertension risk,” *Environment International*, vol. 124, pp. 441-447, 2019.	Not reporting relations with atherosclerosis and/or CAD
52	X. Qiao, S. Bhave, L. Swain, E. Zweck, L. Reyelt, P. Crowley, S. K. Annamalai, A. Chennjorwala, M. Esposito, A. Razavi, S. Foroutanjazi, C. Machen, K. Thayer, L. Korde, R. H. Karas, and N. K. Kapur, “Myocardial Injury Promotes Matrix Metalloproteinase-9 Activity in the Renal Cortex in Preclinical Models of Acute Myocardial Infarction,” *Journal of Cardiovascular Translational Research*, vol. 15(2), pp. 207–216, 2022.	Not reporting MMP-2
53	Y. Chen, A. B. Waqar, K. Nishijima, B. Ning, S. Kitajima, F. Matsuhisa, L. Chen, E. Liu, T. Koike, Y. Yu, J. Zhang, Y. E. Chen, H. Sun, J. Liang, and J. Fan. “Macrophage-derived MMP-9 enhances the progression of atherosclerotic lesions and vascular calcification in transgenic rabbits,” *Journal of Cellular and Molecular Medicine*, vol. 24(7), pp. 4261-4274, 2020.	Not reporting MMP-2
54	Y. Hu, X. Dong, T. Zhang, H. Ma, W. Yang, Y. Wang, P. Liu, and Y. Chen. “Kai-Xin-San suppresses matrix metalloproteinases and myocardial apoptosis in rats with myocardial infarction and depression,” *Molecular Medicine Reports*, vol. 21(1), pp. 508-516, 2020.	An *in vivo* study
55	Y. S. Ma, Y. H. Xie, D. Ma, J. J. Zhang, and H. J. Liu, “Shear stress-induced MMP1 and PDE2A expressions in coronary atherosclerosis,” *Bratislava Medical Journal*, vol. 122(4), pp. 287-292, 2021.	Not reporting MMP-2
56	Y. Yang, G. Li, and R. Zhang, “Correlation Analysis of Acute Coronary Syndrome with Serum IL-18, MMP-9, hs-CRP, and Plasma FIB,” *BioMed Research International*, vol. 2022, pp. 5984184, 2022.	Not reporting MMP-2

**Table 4:** Summary of the findings of the selected studies.

	Study design and subject's characteristics	Mean age (years)	Methods and MMPs measured	MMP-2 level in atherosclerotic CAD	NOS score
Mogharrabi et al. [33]	Case–control, double-blind, randomized clinical trial. 70 patients with CAD (40%–50% stenosis) were randomly assigned into two groups: (i) nanocurcumin group (given nanomicelle 80 mg/day) (ii) control group (given placebo) Treatment was given for 3 months	>18 years old	The activity and expression of MMP-2 and MMP-9 in the serum were measured using RT–PCR and zymography analysis	MMP-2 mean relative gelatinase activity was significantly decreased in CAD patients treated with curcumin compared with the placebo group (*p* < 0.001)	7
Sai et al. [34]	Case–control clinical trial. 56 patients with AMI were randomly divided into: (i) study group (28 patients; 15 men and 13 women): given 20 mg rosuvastatin and 2.5 mg benazepril daily for 3 months. (ii) control group (28 patients; 14 men and 14 women): given 20 mg rosuvastatin daily for 3 months. 30 healthy volunteers were assigned as normal controls (18 men and 12 women)	Study group: 53 ± 12 years old Control group: 54 ± 0.8 years old Normal control: 51 ± 1.2 years old	Serum levels of MMP-2, MMP-9, and leukotriene B4 pre- and posttreatment were measured using ELISA	Serum levels of MMP-2 were significantly higher in AMI patients compared with the healthy subjects (*p* < 0.01). Serum levels of MMP-2 were significantly decreased in AMI patients treated with both benazepril and rosuvastatin compared with rosuvastatin alone (*p* < 0.05)	8
Li et al. [35]	Case–control study 80 patients with acute coronary syndrome: (i) acute group (40 patients; 19 male and 21 female, 26 AMI patients and 14 UAP patients, course of disease ranged from 1 to 6 years) (ii) stable group (40 patients; 18 male and 22 female, 25 AMI patients and 15 UAP patients. course of disease ranged from 2 to 8 years) 40 healthy subjects (control group)	Acute group (37–73 years; mean age: 53.27 ± 1.45 years) Stable group (39–71 years; mean age: 53.04 ± 1.38 years) Control group (37–68 years; mean age: 52.85 ± 1.46 years)	Serum MMP-2 levels were measured using ELISA	MMP-2 levels were significantly higher: (i) in the acute and stable groups compared with the control group (*p* < 0.05) (ii) in the acute group compared with the stable group (*p* < 0.05) (iii) in the AMI patients compared with the UAP patients (*p* < 0.01)	9
Murashov et al. [36]	Cross-sectional study 68 men with coronary atherosclerosis who underwent coronary bypass surgery with endarterectomy	46–79 years old	MMP-2 expression in the stable (*n* = 21) and unstable (*n* = 31) atherosclerotic plaque in the coronary arteries was determined using immunohistochemistry (IHC)	Expression of MMP-2 in the unstable atherosclerotic plaque was 7.8 times higher in comparison with the stable atherosclerotic plaque (*p* < 0.05). MMP-2 was mostly expressed in the cytoplasm of foamy macrophages in the atheromatous core and in the caps of unstable plaque with lipid erosions	4
Murashov et al. [37]	Cross-sectional study 33 men with occlusive coronary atherosclerosis who underwent coronary bypass surgery with endarterectomy	62.5 ± 10.9 years	MMP-2 expression was measured using IHC in different types of unstable coronary artery plaques, namely necrotic-degenerative type (64%), lipid type (23%), and inflammatory-erosive type (13%)	No significant difference in MMP-2 expression among the three different types of unstable atherosclerotic plaque, suggesting that accumulation of MMP-2 was present in all types of unstable plaque	5
Melin et al. [38]	Cross-sectional study 268 Type 1 diabetes (TID) patients with CVD, depression, thyroid disease, hypertension, and hyperlipidemia	18–59 years old	Plasma levels of MMP-2, MMP-14, TIMP-2, and TIMP-3 were analyzed by ELISA	MMP-2 and CVD were independently associated with high levels of MMP-14 in T1D patients	5
Malkani et al. [39]	Case–control study 200 Pakistani subjects (i) 100 patients with coronary atherosclerosis (ii) 100 healthy controls	NS	Genomic DNA was extracted from blood samples and subjected to RFLP-PCR analysis for two SNPs of the MMP-2 gene (rs 243865 and rs 243866)	Both allelic and genotype frequencies of rs243865 were higher in atherosclerosis patients than the healthy controls (*p* < 0.01) Only allelic frequency of rs243866 was higher in atherosclerosis patients than the healthy controls (*p* < 0.01) Haplotype analysis indicated that CA, CG, and TA haplotypes of the MMP-2 gene were significantly connected with atherosclerosis (*p* < 0.01)	7
Owolabi et al. [40]	Cohort study 64 subjects with AMI and stable CAD were divided into two main groups: (i) stable CAD (*n* = 15) (ii) acute MI (*n* = 49), which was further divided into atherothrombotic MI (*n* = 22) and non atherothrombotic MI (*n* = 12)	>18 years old	Plasma MMP-2 levels were measured using multiplex immunoassay at two main time points: (i) acute phase (at the start of cardiac catheterization and 6 hr post catheterization) (ii) quiescent phase (at 3 months follow-up after acute phase)	No significant difference in MMP-2 levels between acute MI vs. stable CAD, and atherothrombotic vs. nonatherothrombotic MI at any time point	9

AMI, acute myocardial infarction; CAD, coronary artery disease; CVD, cardiovascular disease; ELISA, enzyme-linked immunosorbent assay; MI, myocardial infarction; MMP, matrix metalloproteinase; NS, not stated; RT–PCR, reverse transcription–polymerase chain reaction; RFLP–PCR, restriction fragment length polymorphismpolymerase chain reaction; TIMP, tissue inhibitor of matrix metalloproteinase,; UAP = unstable angina pectoris.

**Table 5:** List of sources of funding for studies included in the review.

Included studies	Sources of funding
Mogharrabi et al. [33]	Mashhad University of Medical Sciences Research Council
Sai et al. [34]	Not stated
Li et al. [35]	Not stated
Murashov et al. [36]	Not stated
Murashov et al. [37]	Not stated
Melin et al. [38]	Research and Development Fund of Region Kronoberg, Växjö, Sweden, and by the Research Council of South Eastern Sweden (FORSS), Linköping, Sweden, and the Southern Healthcare Region, Lund, Sweden
Malkani et al. [39]	Not stated
Owolabi et al. [40]	American Heart Association (11CRP7300003), and National Institutes of Health (1P20 GM103492)
